# Multicentric Osteolysis Nodulosis and Arthropathy (MONA): A Case Series and Review of the Literature

**DOI:** 10.31138/mjr.311203.mon

**Published:** 2024-07-24

**Authors:** Mahabaleshwar Mamadapur, Sabarinath Mahadevan, Ponniah Subramanian ArulRajamurugan, Srilakshmi Gandham, Swati Singh

**Affiliations:** 1 Department of Clinical Immunology and Rheumatology, JSS Medical College and Hospital, JSS Academy of Higher Research, Mysore, Karnataka, India; 2 O.M. HEALTH CARE, Tamilnadu, India; 3 Madras Medical College, Chennai, Tamil Nadu, India; 4 Kasturba Medical College Manipal, Manipal Academy of Higher Education, Manipal, Karnataka, India; 5 Department of Medical Genetics, Kasturba Medical College, Manipal Academy of Higher Education, Manipal, Karnataka, India

**Keywords:** MMP2, osteolysis, Torg Syndrome, MONA syndrome, juvenile idiopathic arthritis

## Abstract

Multicentric Osteolysis Nodulosis and Arthropathy (MONA) is a rare skeletal disorder driven by mutations in the MMP2 gene, leading to bone and joint degradation. This case series presents three unique MONA cases, highlighting clinical, radiological, and genetic aspects. These insights shed light on the complexities of MONA, aiding early diagnosis and multidisciplinary management.

Case 1 is a 13-year-old male, born to consanguineous parents, presented with a 5-year history of progressive joint deformities, pain, and difficulty walking. Initially diagnosed as juvenile idiopathic arthritis (JIA), despite treatment, his symptoms persisted. Examination revealed multiple clinical findings, including joint contractures and nodules. Genetic analysis identified a pathogenic variant in the MMP2 gene, confirming MONA. Case 2 and Case 3 were two siblings, aged 12 and 17 years respectively, who presented progressive joint contractures in their hands and feet since early childhood. Clinical examinations revealed contractures and subcutaneous nodules. Genetic analysis confirmed MONA with a shared homozygous pathogenic MMP2 variant, emphasising the genetic basis of this rare disorder.

## INTRODUCTION

Multicentric Osteolysis Nodulosis and Arthropathy (MONA), also known as Torg-Winchester Syndrome, is a rare and debilitating skeletal disorder characterised by progressive osteolysis, joint abnormalities, and the development of subcutaneous nodules. Other manifestations include pigmented skin lesions, cardiac defects, coarse facies, corneal opacities, and gum hypertrophy. The age of onset can range from birth to 11 years. First described by Torg and Winchester in 1971, this condition remains poorly understood due to its extreme rarity and limited case reports in the medical literature.^1^ There have been documented cases of 51 patients, hailing from 31 distinct families, exhibiting 24 distinct pathogenic variants within the MMP2 gene.^2,3^ These cases have been reported across various regions, including Saudi Arabia, Turkey, India, Egypt, Brazil, South America, Korea, Italy, Finland, Greece, Algeria, and Morocco.^4^

The pathogenesis of MONA revolves around mutations in the matrix metalloproteinase 2 (MMP2) gene, which encodes for a crucial enzyme involved in extracellular matrix remodelling. Dysfunction of MMP2 leads to abnormal collagen synthesis, resulting in the progressive degradation of bone and joint structures.^5^ Consequently, MONA patients typically present with severe musculoskeletal manifestations, including skeletal deformities, joint contractures, and chronic pain, significantly compromising their quality of life.^6^

This case series aims to contribute to the existing knowledge regarding MONA by presenting three unique and detailed cases. These cases offer valuable insights into the clinical presentation, radiological findings, genetic mutations, and therapeutic approaches associated with this rare disorder. By shedding light on the complexities of MONA, we aim to improve early recognition, diagnosis, and management of affected individuals. Furthermore, we discuss the challenges in managing MONA, including the limited treatment options and the need for a multidisciplinary approach involving rheumatologists, orthopaedic surgeons, geneticists, and physical therapists.

## CASE REPORTS

### Case 1

**Figure 1.**
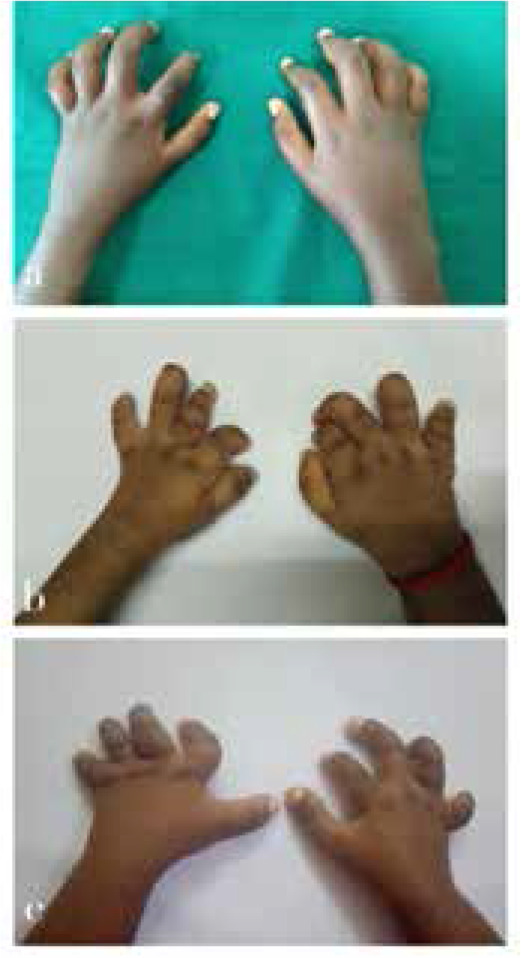
Hands of patients show joint contractures and swelling of digits. (A) P1 at 12 years. (B) P2 at 12 years, (C) P3 at 17 years. Camptodactyly can be seen in P1. Resorption of underlying bones causes shortening of digits and excess skin.

**Figure 2.**
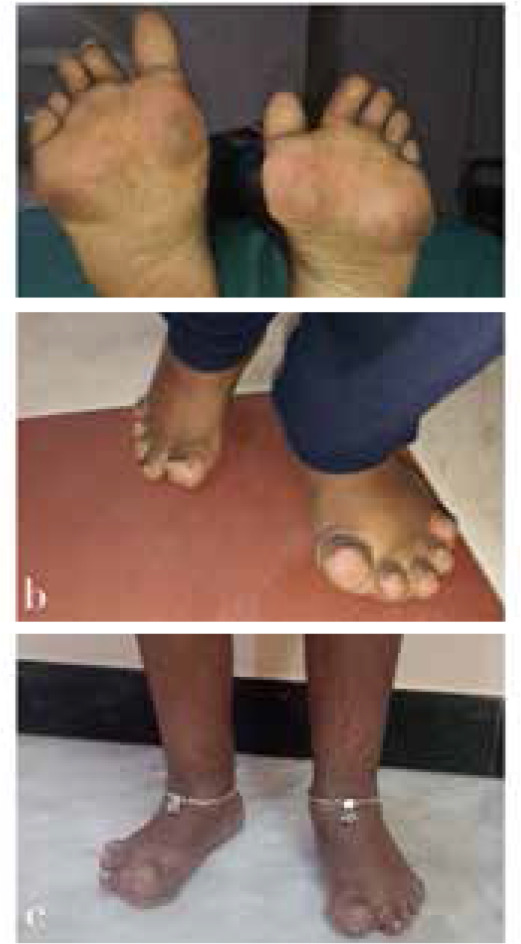
Hands of patients show joint contractures and swelling of digits. (A) P1 at 12 years. (B) P2 at 12 years, (C) P3 at 17 years. Camptodactyly can be seen in P1. Resorption of underlying bones causes shortening of digits and excess skin.

A 13-year-old male, born to consanguineous parents, presented to our paediatric rheumatology clinic with a history of progressive deformities in his hands and feet, as well as difficulty in walking over the past five years. Five years ago, the patient’s symptoms began with swelling and pain in the left 3rd proximal interphalangeal (PIP) joint. Subsequently, he noticed the development of plantar nodules and swelling in wrists, knees, and ankles. During the initial stages of his illness, he sought medical attention primarily for joint pain and swelling and received symptomatic treatment. Approximately two years before his presentation to our clinic, he was diagnosed with juvenile idiopathic arthritis, enthesitis related arthritis (JIA-ERA). Despite being prescribed disease-modifying antirheumatic drugs (DMARDs) and biologics, there was limited improvement. Upon examination, he had coarse facies, antalgic gait, contractures in the hands with swelling of the digits, fixed flexion deformity at both elbows, restricted hip movements in flexion and abduction, and bilateral knee joint line tenderness. His spine examination was normal, but soft to firm nodules, each measuring approximately 2.5 x 2.5 cm, were observed in both feet and elbows **(Figures 1** and **2)**.

**Figure 3.**
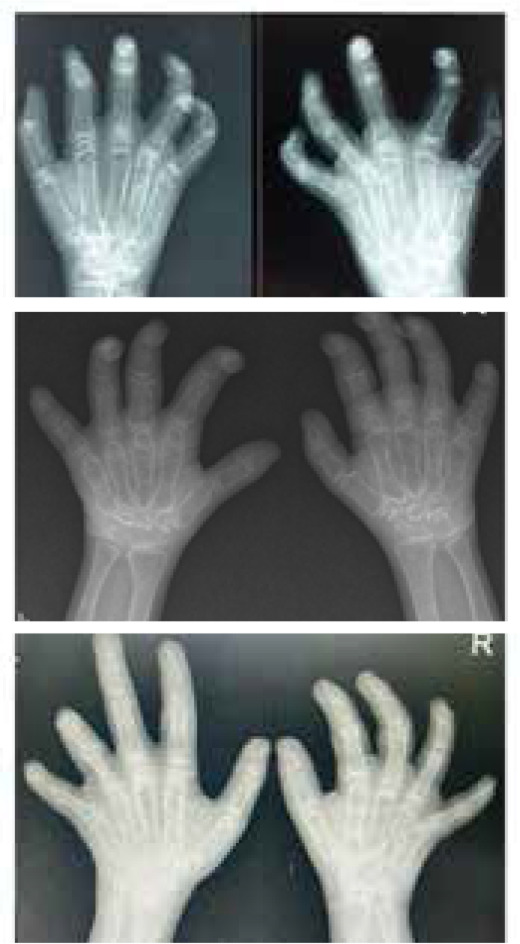
Hand radiographs of children with MONA. (A) P1 at the age of 12 years. (B) P2 at the age of 5 years. (C) P3 at 10 years. well-formed carpals can be seen in the early stages (A) and (C). In the later stages diffuse osteopenia, osteolysis of carpal bones, ankylosis of carpal bones, and erosions at the base of PIP can be seen (B).

On evaluation, his haemoglobin was 11.9 gm/dl, and total leucocyte count was 6900 cells/cumm. CRP was 0.3mg/dl. Rheumatoid factor (RF), anti-cyclic citrullinated peptide antibodies (ACPA), and anti-nuclear antibodies (ANA) were normal. 2D ECHO was normal. X-ray findings are summarised in **Figure 3**.

Genetic Analysis identified a homozygous pathogenic variant in exon 2 of the matrix metalloproteinase 2 (MMP2) gene [c.301C>Tp(Arg101Cys)] confirming the diagnosis of Multi-centric Osteolysis Nodulosis and Arthropathy (MONA). The clinical and lab details are mentioned in **Table 1**.

**Table 1.** Distribution of clinical and lab characteristics of patients with MMP2 gene pathogenic variants.

**Patient**	**Patient 1**	**Patient 2**	**Patient 3**
**Gender**	Male	Female	Female
**Consanguinity**	Yes	No	No
**Age at onset**	7 years	3 years	3 years
**Age at presentation**	12 years	12 years	17 years
**Subcutaneous nodules**	Yes	Yes	Yes
**Skin lesions/Hyperpigmentation**	Yes	No	No
**Hirsutism**	No	Yes	Yes
**Coarse face**	Yes	No	No
**Corneal opacity**	NA	No	No
**Nose (bulbous/flat)**	Bulbous	Bulbous	Bulbous
**Gingival Hypertrophy**	No	No	No
**Osteolysis of carpal/tarsal bones**	Yes	Yes	Yes
**Osteolysis of other bones**	Phalangeal joints	Phalangeal joints	Phalangeal joints
**Osteoporosis/osteopenia**	Yes	Yes	Yes
**Wide metacarpals/metatarsals**	Yes	No	No
**Joint contractures**	Yes	Yes	Yes
**Joint swelling**	Yes	Yes	Yes
**Joint stiffness**	No	No	No
**Joint pain**	Yes	Yes	Yes
**Loss of joint space**	Yes	Yes	Yes
**CRP (mg/dl)**	0.3	0.4	0.3
**EKG anomalies**	No	No	No
**Congenital heart defects**	No	No	No
**Other important findings**	Camptodactyly, Subluxation of 1–5 MTP joints, Ankylosis of carpal bones	Subcutaneous swelling over both feet present	Subcutaneous swelling over both feet present
**Mutation**	c.301C>T p(Arg101Cys)	c.380G>A p.Arg127Lys	c.380G>A p.Arg127Lys

### Case 2 and Case 3

#### Patient Information

Two siblings, aged 12 and 17 years respectively, were brought to the paediatric rheumatology clinic due to a shared medical history of progressive contractures in the small joints of their hands and feet, which had been ongoing since early childhood. A thorough clinical examination was conducted for both siblings, utilising Human Phenotype Ontology (HPO) terminology to describe their clinical findings **(Table 1)**. RF, ACPA, and ANA were negative 2D Echo was normal. X-ray findings are summarised in **Figure 3**.

Sanger sequencing identified a homozygous pathogenic variant in exon 2 of the matrix metalloproteinase 2 (MMP2) gene [c.380G>Ap.Arg127Lys] suggestive of Multicentric Osteolysis Nodulosis and Arthropathy (MONA).

## DISCUSSION

Torg Syndrome, Winchester Syndrome, Frank-Ter Haar Syndrome, and MONA are now considered the same spectrum of multisystem disorders since they have significant phenotypical overlap in the form of skin, joint, bone, and heart involvement. Homozygous mutations identified in SH3PXD2B, MMP14, or MMP2 genes responsible for collagen remodelling are responsible for these syndromes.^7–9^ The severity and disease onset may vary. Torg syndrome has milder phenotypic features compared to Winchester syndrome like absence of severe coarse facies, corneal opacities, cardiac symptoms, and severe vertebral involvement.^10^

The clinical features may overlap with Juvenile Idiopathic arthritis, Multicentric Carpal osteolysis with or without nephropathy, Hyaline Fibromatosis Syndrome, and Mucopolysaccharidosis resulting in delayed diagnosis.

The presented cases of Multicentric Osteolysis Nodulosis and Arthropathy (MONA) in a male patient and two siblings highlight the clinical complexity and diagnostic challenges associated with this exceptionally rare autosomal recessive disorder. These cases underscore the importance of accurate diagnosis and genetic evaluation in such cases.

To enhance our understanding of these cases, we reviewed the available literature and found several relevant studies that provide insights into MONA. In a study by Imam et al. (2023), the authors reported an adolescent female with MONA, initially managed as JIA, highlighting the importance of early genetic diagnosis, and preventing unnecessary pharmacological intervention.^11^ In another case series by Ishaq et al. (2023) four patients from two families in Pakistan and Finland revealed consistent MONA features. Genetic analysis identified novel MMP2 gene variants associated with the condition. The above case scenario highlights the potential variations in age of onset and differences in mutations in the same family.^12^

Castberg et al. (2013) reported a 5-year-old boy with MONA and congenital heart defects. Despite treatment efforts, the patient experienced ongoing bone degeneration. This case highlights the natural progression of MONA and its potential association with cardiac issues, suggesting that cardiac evaluations be considered for all MONA patients.^13^ Bhavani et al. (2015) conducted a comprehensive screening of MMP2 mutations in thirteen individuals from eleven families, unveiling eight mutations—five novel and three known variants.^4^ If MMP2 pathogenic variants are identified in a family member with MONA, carrier testing can be conducted for at-risk relatives. Additionally, for pregnancies at an increased risk, prenatal and preimplantation genetic testing are viable options to assess the presence of pathogenic variants.

In a case study presented by Shakiba et al. (2023), a 5-year-old girl, the first child of consanguineous parents, exhibited progressive limb deformity, poor growth, and bone pain. Physical examination revealed facial dysmorphism, hypertrichosis, severe hand deformity with limited joint mobility, hallux valgus deformity in the feet, soft tissue hypertrophy, and nodules in palmoplantar areas. Genetic analysis revealed a novel homozygous mutation in the MMP2 gene.^6^

Treatment options are limited and are mainly supportive. Calcium and vitamin D supplementation may benefit to some extent. At present there is no specific therapy that can prevent the progression of osteopenia and osteolysis. steroids and immunosuppressants are less effective and better avoided. Bisphosphonate therapy decreased bone pain, and improved bone mineralisation in a few case reports. However, there was no improvement in joint motion. Early initiation of treatment can achieve the best outcomes.^14,15^ Physical therapy helps to slow the rate of development of contractures. The role of surgical release of contractures is variable. Walking aids may be needed as the disease progresses.

In conclusion, MONA remains an exceptionally rare skeletal dysplasia with a complex clinical presentation, making it crucial for healthcare providers to maintain an index of high suspicion in cases of progressive joint contractures with skin, bone, heart, eye involvement, and related symptoms, normal inflammatory markers and which do not respond to conventional treatment, particularly in consanguineous families. Early genetic evaluation is essential for achieving a precise diagnosis, enabling appropriate medical management, and providing the necessary support to affected individuals and their families. These cases contribute to our understanding of MONA and remind us of the importance of comprehensive genetic assessment in cases of rare musculoskeletal disorders.
